# Structural Evolution and Magnetic Properties of Gd_2_Hf_2_O_7_ Nanocrystals: Computational and Experimental Investigations

**DOI:** 10.3390/molecules25204847

**Published:** 2020-10-21

**Authors:** Madhab Pokhrel, Nicholas Dimakis, Chamath Dannangoda, Santosh K. Gupta, Karen S. Martirosyan, Yuanbing Mao

**Affiliations:** 1 Department of Physics and Astronomy, University of Texas Rio Grande Valley, 1201 W University Drive, Edinburg, TX 78539, USA; chamath.dannangoda@gmail.com (C.D.); karen.martirosyan@utrgv.edu (K.S.M.); 2 Radiochemistry Division, Bhabha Atomic Research Centre, Trombay, Mumbai 400085, India; santoshg@barc.gov.in; 3 Department of Chemistry, Illinois Institute of Technology, 3105 South Dearborn Street, Chicago, IL 60616, USA

**Keywords:** Gd_2_Hf_2_O_7_, pyrochlore, disorder fluorite, structural evolution, molten-salt synthesis, DFT

## Abstract

Structural evolution in functional materials is a physicochemical phenomenon, which is important from a fundamental study point of view and for its applications in magnetism, catalysis, and nuclear waste immobilization. In this study, we used x-ray diffraction and Raman spectroscopy to examine the Gd_2_Hf_2_O_7_ (GHO) pyrochlore, and we showed that it underwent a thermally induced crystalline phase evolution. Superconducting quantum interference device measurements were carried out on both the weakly ordered pyrochlore and the fully ordered phases. These measurements suggest a weak magnetism for both pyrochlore phases. Spin density calculations showed that the Gd^3+^ ion has a major contribution to the fully ordered pyrochlore magnetic behavior and its cation antisite. The origin of the Gd magnetism is due to the concomitant shift of its spin-up 4f orbital states above the Fermi energy and its spin-down states below the Fermi energy. This picture is in contrast to the familiar Stoner model used in magnetism. The ordered pyrochlore GHO is antiferromagnetic, whereas its antisite is ferromagnetic. The localization of the Gd-4f orbitals is also indicative of weak magnetism. Chemical bonding was analyzed via overlap population calculations: These analyses indicate that Hf-Gd and Gd-O covalent interactions are destabilizing, and thus, the stabilities of these bonds are due to ionic interactions. Our combined experimental and computational analyses on the technologically important pyrochlore materials provide a basic understanding of their structure, bonding properties, and magnetic behaviors.

## 1. Introduction

Structural disorders have a profound influence on a material’s properties, and lead to the development of highly advanced technological applications, such as 2D-ferromagnets, light-emitting devices, catalysts, electronic conductors, etc. [1,2,3,4]. Primarily, phonon, electron, and heat transport properties are strongly influenced by crystallographic defects and, thus, they can be found in diverse technological applications [1,2,3,4]. For example, electron transport properties in graphene, photon transport in silicon photonics, heat transfer in thermoelectric materials, and photoluminescence yield in luminescence materials are altered due to structural disorder [5,6,7,8,9,10,11]. Specifically, in technologically important materials, such as A_2_B_2_O_7_ pyrochlores, structural disorder and point defects play a decisive role in their catalytic activity, ionic conductivity, and radiation stabilities [12,13,14,15,16].

The structural stabilities of the A_2_B_2_O_7_ (ABO) crystalline phases are guided by the A and B ionic radii ratio: If r_A_/r_B_ < 1.46, the defect fluorite (DF; Fm $\underline{3}$ m space group) is favored over the ordered pyrochlore (OP; Fd $\underline{3}$ m space group) [12,14]. On the other hand, if r_A_/r_B_ > 1.80, the ABO yields monoclinic phases (noncubic pyrochlore) [17]. This thermodynamic process depends on the energies required for defect formation and increases along with an increasing ionic radii ratio (r_A_/r_B_) [12,18,19]. Macroscopically, the ideal fluorite has a structure similar to a mineral fluorite structure such as CaF_2_, with one cation and one anion site. However, recent studies showed that the DF structure is distorted at the nanoscale [20]. Zhang et al., using synchrotron-based x-ray diffraction and absorption measurements, reported that the pyrochlore local disorder exists before the detection of the fluorite phase boundary and that it continues throughout the DF region [21].

Shamblin et al. have provided a deep insight into the DF structure using neutron scattering measurements [7]. They have proposed a DF structure of local orthorhombic structural units with orthorhombic and isometric arrays in a repetitive pseudo-translational symmetry [7]. Both cations share the seven oxygen atoms in the DF structure, where cations and anions/vacancies are fully ordered in the cubic OP structures. The A-site cation is coordinated with eight oxygens, and the B site cations are coordinated with six oxygens, which suggests that vacancies are localized around the B site cation. The difference in the A and B sites’ coordination with oxygen and vacancies causes the OP unit cells to double their size relative to that of DF [7].

Among various ABO compounds, the Gd_2_Hf_2_O_7_ (GHO) stands out, because of its interesting properties, such as its wide temperature range for phase stability, high dielectric constant, and melting point. These properties allow the GHO to serve in broad applications, such as magnetic materials, dielectric coatings, high-temperature ceramics, solid electrolytes in oxide fuel cells, and thermal barrier coatings [9,22,23,24,25]. Interestingly, GHO r_Gd_/r_Hf_ ~ 1.48, which is slightly greater than 1.46, and thus offers a unique opportunity to study the DF or weakly ordered (WO) to OP phase evolution, as this can be induced thermodynamically [26]. Apparently, a vast majority of studies are dedicated to the OP-GHO prepared by solid-state reaction methods, which are usually carried out at high temperature (>1300 °C) and produce the OP-GHO phase directly [27,28,29,30,31]. This kind of high-temperature synthesis method doesn’t allow for the study of the WO-OP crystalline phase evolution of the GHO nanocrystal structure thermodynamically, especially when samples are calcined from lower to higher temperatures. Although the exact lower boundary temperature for the WO-OP crystalline phase evolution is still under debate, it has been reported that GHO stabilizes into fully ordered GHO at ≥1300 °C [26,32]. The ordering of anions, cations, and vacancies on a single site occurs simultaneously in order to achieve the OP-GHO structural transformation. However, it is still unclear how these local orders arise and how they are related to the DF, WO, and OP structures.

Although there are few articles on GHO synthesis and optical properties, these have not been sufficiently exploited. Thus, the understanding of the GHO ordering mechanism is still incomplete [7,33,34,35]. Several recent studies pointed to the importance of synthesizing nanocrystals with a controlled size, shape, and crystalline phase. We recently showed how GHO and other ABO (A = Y, La, Pr, Er, and Lu, B = Hf, and Zr) compounds can be synthesized in the nanodomain, using the molten salt method. The functionality of such nanocrystals depends on the crystalline phase and local structure [32,36,37]. It should be noted that the GHO morphology and crystalline size can be altered by varying the synthesis condition and annealing temperature [32]. In recent years, we have investigated the molten-salt synthesis of the A_2_B_2_O_7_ (A = Y, La, Pr, Gd, Er, and Lu, B = Hf, and Zr) nanocrystals and explored their unique set of optical properties when doped with trivalent rare-earth ions and actinides, such as uranium [11,32,38,39]. The molten-salt synthesis has the advantage of homogenizing the binary cations within the complex precursor. Samples are prepared using the coprecipitation method, which allows for the formation of single-phase ABO nanocrystals just above the eutectic point [40,41]. With the molten salt synthesis method, we have previously shown that homogeneous ABO (A = Y, La, Pr, Gd, Er, and Lu; B = Hf, Zr) powders can be synthesized with a crystalline size <30 nm and that the diffraction patterns are similar to those observed in the DF and OP phases depending on the ionic radius ratio rules. Thus, we found the molten-salt synthesis method to be appropriate for producing homogeneous GHO nanocrystals and probing the evolution of their crystalline phases [32,37]. In addition, an understanding of the origin of the crystalline phase evolution is missing thermodynamically, especially when the low-temperature-stabilized GHO nanocrystals are calcined to higher temperatures compared to that of the order-disorder (O-D) transition under high-energy radiation exposure. It is important to mention that the OP-ABO (A = La; B = Zr and Ti) have been extensively studied as a matrix for nuclear waste immobilization [13,34,35,42,43,44]. In this paper, we use the molten-salt synthesis method, where we have stabilized the GHO homogeneous nanocrystals (≈19 nm) in the WO-GHO phase at 650 °C and the OP-GHO phase at 1250 °C.

In the periodic table, the Gd^3+^ ion has the maximum number of seven unpaired electrons and donates 6s^2^ and 5d^1^ electrons to bonding, leaving the highly localized 4f subshell intact, which accounts for the Gd^3+^ weak ferromagnetism [45]. It is reported that Gd metal nanocrystals displayed the highest magnetization that had been measured to date, with 206 emu/g recorded at 2 K [46]. However, such information is completely lacking in the GHO, which goes through the crystalline phase evolution thermodynamically. Moreover, information on the magnetic moment in both WO-GHO and OP-GHO phases is important in order to understand the spin origin in this material, which is needed in the design of magnetic materials. Thus, we carried out magnetic measurements on both WO-GHO and OP-GHO. In addition, we performed electronic calculations using first principles to investigate the source of magnetism in the OP-GHO structure.

This work reports on the thermally induced phase transition in GHO from weakly ordered pyrochlore to ordered pyrochlore. Moreover, we have done extensive density functional theory (DFT) [47,48] calculations to explore the structural and electronic properties of the OP-GHO and its antisite. Such an analysis predicted the weakly ferromagnetic insulating characteristics of the GHO antisite. The local chemical bonding information in GHO NPs is also predicted based on Crystal Orbital Overlap Populations (COOP) [49,50] and Crystal Overlap Hamilton Populations (COHP) calculations [51]. The COOP results from multiplying the densities-of-states (DOS) by the overlap population: It provides bonding information between two atoms. Positive and negative COOP values are indicative of stabilizing and destabilizing covalent interactions, respectively (-COHP is equivalent to COOP). Overlap population values close to 0 correspond to noncovalent interactions.

## 2. Experimental Section

### 2.1. Synthesis Procedure

The starting materials include the rare earth (RE) nitrate hexahydrate (Gd(NO_3_)_3_•6H_2_O, analytical grade, 99.0%), hafnium dichloride oxide (HfOCl_2_•xH_2_O, 99.9%), potassium nitrate (KNO_3_, 99.9%), sodium nitrate (NaNO_3_, 98%), and ammonium hydroxide (NH_4_OH, 28.0-30.0%). All materials were purchased from Sigma Aldrich and used without further purification.

Gadolinium hafnate nanocrystals (NCs) were synthesized following a previously reported method [11,32,38,39]. More specifically, the synthesis of GHO follows the two-step process. First, the single-source complex precursor of Gd(OH)_3_·HfO(OH)_2_·nH_2_O) was synthesized via a coprecipitation route. Then, the size-controlled GHO NCs were synthesized through a facile molten salt synthetic process using the single-source complex precursors of Gd(OH)_3_·HfO(OH)_2_·nH_2_O) combined with a nitrate mixture (NaNO_3_:KNO_3_ = 1:1, molar ratio) and annealed at 650 °C for six hours. For more synthesis details regarding these kinds of binary (RE_2_Hf_2_O_7_) oxides, see these references [10,32]. The as-prepared GHO powder was calcined to 1000 °C, 1250 °C, and 1500 °C for six hours in the air (ESI-1).

### 2.2. Sample Characterization

X-ray diffraction (XRD) and Raman spectroscopy (ESI-2) were utilized to evaluate the crystalline phase of as-synthesized and calcinated compounds under different temperatures. The magnetic measurements (M vs. H) of the GHO nanocrystals in both DF and OP phases were measured using the Physical Property Measurement System (PPMS Evercool-2, Quantum Design, Inc.) through a vibrating sample magnetometer (VSM) in the applied field ranges of ± 9 Tesla at 300 K. Scanning Electron Microscopy (SEM) characterization was used (ESI-1) to evaluate the morphology and particle size of the samples (ESI-1).

### 2.3. Computational Methodology

The electronic and structural properties of the Gd_2_Hf_2_O_7_ structure and their corresponding antisites are calculated using the CRYSTAL17 DFT code with Gaussian basis sets centered at the atoms [52]. Spin unrestricted DFT is used here, under the screened hybrid functional HSE06 of Heyd, Scuseria, and Ernzerhof, which provide improved band gaps [47,53,54,55]. Long range interactions are treated using the D3 correction by Grimme et al. [56]. CRYSTAL17′s limitation is the absence of spin-orbit coupling calculations. However, it has been reported that spin-orbit coupling mostly affects the band structure, whereas its effect in optimal geometry is minimal [57,58,59]. Atomic basis sets are optimized for crystalline calculations. The innermost Gd and Hf orbitals are described by the Stuttgart–Dresden effective core potentials (ECP), which account for mass-velocity and Darwin relativistic corrections [60,61]. The Gd 4f orbitals are described in the valence by three Gaussians (i.e., “(3f)” notation). The Gd effective valence basis set is [5(sp)2d3f] and contains 36 electrons [62,63]. Moreover, the Hf effective valence basis set is [3(sp)2d] and contains 12 electrons [64]. The O all-electron basis set [1s3(sp)2d1f] is used [65]. Details regarding the Brillouin zone integrations grids can be found in the Supplementary Materials. SCF energy convergence and geometry optimization parameters are described in our past work [66,67].

Geometries and electron and spin densities are visualized using VESTA [68]. The COOP and COHP overlap populations are available in CRYSTAL17 [49,69]. The COHP accurately accounts for bond strengths by partitioning the band energies, instead of the electron states, into bonding and antibonding regions. Thus, COHP is preferred over COOP. Both COOP and COHP are basis-set-dependent calculations. Grechnev et al. stated that this is due to the possible linear dependencies in the basis sets used for solid state calculations, [70] and thus should not serve as an absolute bonding indicator. The atomic gross populations are calculated using the Mulliken population analysis [71].

## 3. Results and Discussion

### 3.1. Modeling of the OP-GHO Structures

**Figure 1:**
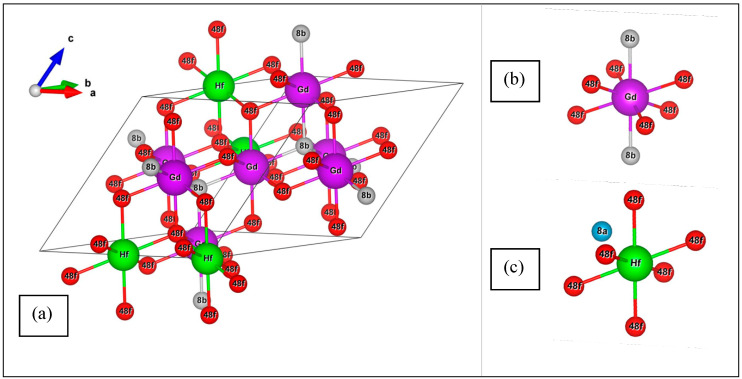
The OP-GHO structure (**a**) primitive cell. Lines show the unit cell boundaries. The O_48f_ and O_8b_ are shown by red and gray spheres, respectively. (**b**,**c**) show the OP-GHO Gd and Hf sites illustrating the O site and its vacancy (O_8a_; cyan sphere).

The OP-GHO structures are modeled as cubic Fd $\bar{3}$ m lattices (space group #227), in agreement with experimental observations [25,26,72,73]. Figure 1 shows the primitive OP-GHO unit crystal structure, which is triclinic. As stated above, the Gd and Hf atoms occupy the sites 16*d* and 16*c*, respectively [74]. Moreover, six oxygen (O) atoms occupy the O_48f_ site (*x*, 1/8, 1/8), where *x* is the internal structural parameter. The remaining O atom occupies the O_8b_ site (3/8, 3/8, 3/8). The O vacancy site (O_8a_), which is located at (1/8, 1/8, 1/8), is simulated via two different methods: 1) placing a massless “ghost” O atom in the O_8a_ site, which has the same basis set as any other O atom in the pyrochlore and 2) leaving the O_8a_ site vacant. The O ghost atom has the same basis set as any other oxygen atoms in the structure. Ghost atoms have been used for simulating electrons trapped at a negative-ion vacancy within the crystal (F-centers) [75,76] and trapped-hole centers in alkaline earth oxides [77]. Malia et al. found that ghost atoms provided an improved description of a vacancy relative to having an atom removed from the lattice: Ghost atoms allow charge build-up at the vacancy site [75]. Thus, vacancies simulated via ghost atoms are advantageous relative to simulations with sites that are vacant.

In the cation antisite, the cations swap their positions in the lattice (i.e., in the antisite, Gd and Hf are located at 16*c* (0, 0, 0) and 16*d* (½, ½, ½), respectively) [10]. The OP-GHO unit cell contains 22 atoms when modeled with the O_8a_ site being vacant, and 24 atoms otherwise. The OP-GHO and its antisite antiferromagnetic configurations were explored using larger supercells of 44 atoms. The ferromagnetic antisite was explored by using both the 22 (24 when ghost atoms were used) and 44 atom supercells.

### 3.2. Structural Parameters for the OP-GHO

Table 1 shows the lattice constant a, the internal parameter *x* (lattice parameters), and the nearest Gd-Hf and metal-O interatomic distances for the OP-GHO DFT-optimized geometries and their corresponding cation antisites. Our OP-GHO lattice constant, with the O_8a_ vacancy simulated via ghost atoms, is very close to the one reported from experiments [78]. This value is smaller than corresponding past periodic DFT calculations, which employed generalized gradient approximation (GGA) functionals without long-range corrections [79]. However, our internal parameter x for the OP-GHO is very close to these past calculations (Table 1). Moreover, our nearest Gd-O_8b_, Gd-O_48f_, and Hf-O_48f_ distances are in agreement with Li et al. [74]. We must state that we are not aware of past structural information for the OP-GHO cation antisite.

We compare the geometries of the OP-GHO stable structures, modeled as described above. The presence of the O_8a_ vacancy in the OP-GHO affects both its lattice constant and its internal parameter (Table 1). The OP-GHO lattice constant is larger when the O_8a_ vacancy is simulated via ghost atoms, relative to the case where the O_8a_ site is vacant. Simulating the O_8a_ vacancy via ghost atoms allows for a charge buildup, which is otherwise lost if the basis sets are absent at these sites. The changes in the OP-GHO lattice constants due to the different vacancy simulation approaches mostly affect the Hf-Gd and Hf-O_8b_ distances relative to all other interatomic distances reported in Table 1.

Swapping the cations affects the OP-GHO geometries (Table 1). The OP-GHO cation antisite lattice constant is decreased relative to its value at the OP-GHO, whereas the opposite is observed for its internal parameter *x*. The above-decreased lattice parameter shortens the Hf-Gd distance and affects the nearest metal-O_48f_ distances.

### 3.3. X-Ray Diffraction and SEM Analysis

**Figure 2:**
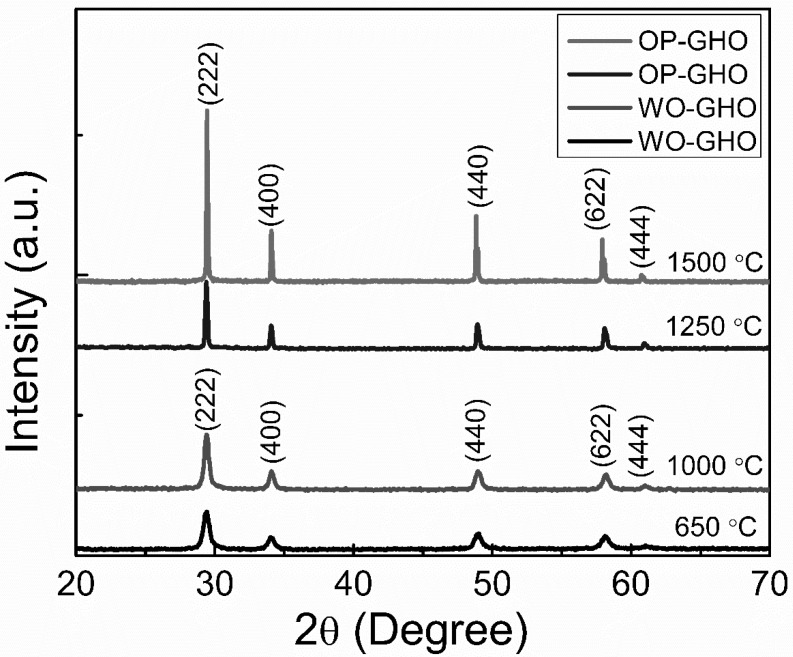
XRD patterns of GHO powders annealed at different temperatures for 6 h.

Powder XRD (Figure 2) and SEM (ESI-1) were used to investigate the crystalline phase, particle size, and morphology of the as-synthesized and calcined samples. It was found that the crystallization of the Gd(OH)_3_·HfO(OH)_2_·nH_2_O) single hydroxide complex precursor to GHO occurred when combined with a nitrate mixture (NaNO_3_:KNO_3_ = 1:1, molar ratio) and annealed at 650 °C for six hours, based on the XRD analysis (Figure 2). In addition, peaks corresponding to secondary impurity phases (Gd_2_O_3_ and HfO_2_) were not observed, signifying that the GHO powder was well crystallized. At 650 °C, the GHO powders appeared to be homogenized and nanocrystalline (≈19 nm) (see ESI-1a), and the XRD pattern was matched with the space group of the $Fd\bar{3}m\;$ pyrochlore structure with JCPDS No. 78-1292. JCPDS stands for Joint Committee on Powder Diffraction Standards. It is the “old name” of our ICDD (International Centre for Diffraction Data).

The as-synthesized particle morphologies were found to be regular and spherical in shape (ESI-1a). The XRD patterns of the obtained GHO powder after calcination to 1000 °C (see size details ESI-1b) were found to be consistent with those of powders obtained at 650 ℃, demonstrating that they had the same crystal structure. These four prominent diffraction peaks, referred to fully ordered pyrochlore peaks, are indexed as (222), (400), (440), and (662), respectively, assuming a pyrochlore unit cell structure. These peaks are not the only acceptable reflections in the angular range between 20–60 °C for an oxide with a pyrochlore crystal structure. Specifically, the absence of superlattice peaks ((311), (331), (511), and (531)) corresponding to 2θ = 27, 36, 43, and 50° reflections indicates that the GHO powder maintains the WO structure even after calcination to 1000 °C. Using solely the XRD results as the phase indicator for our samples, the GHO maintained the WO-GHO phase below the 1000 °C calcination temperature. However, we started noticing some changes in the XRD patterns when the GHO powder was calcined to 1250 °C, where the quantitative change in XRD patterns (Table S1) was observed only after calcining powders at or above 1250 °C. This can be clearly seen in the full width at half maximum (FWHM) values mentioned in Table S1. The SEM images of the calcined powders at 1250 °C and 1500 °C suggests that teh mean crystalline size increases after calcination from ≈19 nm at 650 °C to ≈1.0µm (1250 °C, 6 h) and ≈1.4 µm (1500 °C, 6 h) (ESI-1c and ESI-1d). However, the absence of superlattice peaks after calcining powder to 1500 °C (Figure 2) indicates that the OP structure was still not fully evolved. It is possible that GHO powders calcined at 1250 °C and 1500 °C still crystallize in an ordered pyrochlore phase, though not a perfectly ordered. Few reports exist on high-temperature transformations in similar compounds in the bulk phase, and the existing data shows the discrepancy. The reported phase transformation temperatures are 1530 °C and 2400 °C, for Gd_2_Zr_2_O_7_ and GHO, respectively [7]. In the Gd_2_Zr_2_O_7,_ a cation disorder of 36.3% was reported for a sample calcined at 1277 °C for 24 hours [7]. These phase transition temperatures are different from our observation, but they indicate that in order to obtain perfectly ordered OP-GHO powders with superlattice peaks, our samples should be calcined to a higher temperature for a more extended period than in our present study. This further shows that powder XRD alone may not be a reliable technique to study the phase evolution in these kinds of oxides.

We further calculated the lattice parameter ‘a’ using the XRD peak (222) corresponding to the OP structure (see details in ESI-3). The lattice parameters were calculated to be GHO-650 °C (10.49 Å), GHO-1000 °C (10.49 Å), GHO-1250 °C (10.50 Å), and GHO-1500 °C (10.50 Å). For all tested compositions, the size of the lattice parameter was consistent with the increasing temperature. This calculation was solely done while considering that as-synthesized and calcined samples resembled the OP-GHO structure. The experimental values were in agreement with the computationally calculated values, as shown in Table 1. The average crystallite sizes of the as-synthesized NPs and the powder calcined at 1000 °C were calculated from the XRD data using the Scherer equation and ranged from 19.0 nm to 42 nm (ESI-3).

In summary, we found that it was difficult to observe very weak superstructure peaks on powder samples using regular low-power broad x-ray sources. Since the volume of diffraction domains is linearly dependent on the grain size, we can safely say that all our samples have an extremely smaller grain size (diffraction domains) than that observed for La_2_Zr_2_O_7_ [33,80]. Arai et al. indicated that below the threshold grain size value (100 nm), the diffraction patterns are similar to those observed in DF [80]. As the grain size increases, the valences for Gd^3+^ and Hf^4+^ reach the optimal value, causing an increase in the volume of diffraction domains [33,80]_._ In addition, the broadening of superlattice reflections is important, even in the larger sized particles, in order to observe them in the XRD pattern. The significant increase in the size of the particles for GHO-1250 °C (≈1.0µm) and GHO-1500 °C (≈1.4 µm) and the increase in the crystallinity based on the XRD analysis (Figure 2 and FWHM in Table S1) led us to believe that both GHO-1250 °C and GHO-1500 °C samples went through a full phase evolution. Specifically, the GHO-650 °C and GHO-1000 °C samples were more stabilized in the WO-GHO phase than in the DF phase, although the superlattice reflections were absent in the XRD patterns. Therefore, we further performed a Raman spectroscopic analysis on all samples.

### 3.4. Raman Spectroscopy

**Figure 3:**
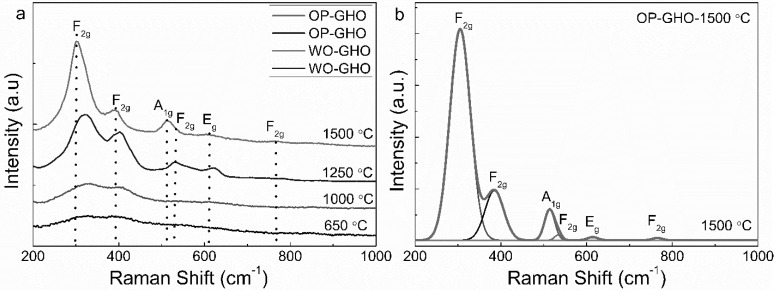
Raman spectra of GHO nanocrystals processed at different temperatures: (**a**) full view; (**b**) the summary of the fitted Raman spectra of the GHO nanocrystals annealed at 1500 °C for 6 h in a box furnace.

Raman spectroscopy has been more widely utilized than XRD to differentiate DF, WO, and OP phases because of its sensitivity. Therefore, we used Raman spectroscopy as an alternative to XRD to examine whether our samples had a thermodynamic phase evolution when the as-synthesized GHO powder was calcined at different temperatures. More specifically, the DF Raman spectra have a single broad-spectrum, since the seven oxygen ions are randomly distributed over the eight anion sites, imposing disorder to the structure. This random distribution of oxygens ensures a broad continuum of densities of states to the Raman spectrum [81]. For as-synthesized GHO powder, two distinct Raman peaks centered at 305 and 406 cm^−1^ support the formation of WO-GHO pyrochlore, matching the XRD analysis of WO-GHO. The Raman spectra of the examined GHO powder calcined at different temperatures are shown in Figure 3a,b. With the calcination of the as-synthesized GHO powder to 1000 °C, Raman peaks at 328 and 406 cm^−1^ are more distinct and pronounced, indicating the crystalline phase evolution in the as-synthesized WO-GHO powders. However, the persistent but more distinct two peaks agree with the appearance of Raman peaks, as expected for evolving WO-GHO. The distinct two peaks clearly indicate that O^2-^ ions are still randomly oriented within the eight anion sites, leading to the high level of structural disordering. The ionic radius ratio of r_A_/r_B_ plays an important role in stabilizing the GHO structure. It has been reported that if r_A_/r_B_ < 1.46, DF is highly likely to form, but that if r_A_/r_B_ >>1.46, a highly ordered pyrochlore (OP) phase is more likely to form. As we discussed previously, GHO exists in the boundary (i.e., r_A_(Gd^3+^)/r_B_ (Hf^4+^) = 1.48) and, thus, a WO phase is more likely to form because reaction kinetics are so slow that ordering is not complete during the low-temperature synthesis.

From the factor group analysis, it is known that the Raman spectra of an ideal cubic OP structure with space group Fd $\underline{3}$ m has six Raman active modes, that is *Γ_pyro_* = *A*_1g_ + *E*_g_ + *4F*_2g_ [81,82]. It is clear from the presence of the six Raman active modes, as shown in Figure 3a, that the GHO undergoes a WO-to-OP phase evolution when the as-synthesized powder is calcined at or above 1250 °C. These observations closely match previous studies on similar compounds and are consistent with Ostwald’s step rule, as it was reported that the OP structure in GHO had a low enthalpy of formation compared to that of WO [29,83,84]. The Gd_2_Hf_2_O_48f_O_8a_ OP structure is composed of five cation-anion vibrations, and the highest Raman mode is related to the oxygen sublattice, as shown in Figure 3a,b [85,86]. When calcined to 1500 °C, the Raman spectra profiles become sharper when compared to those calcined at 1250 °C, indicating a fully ordered pyrochlore phase evolution. Thus, we fitted the Raman spectra of OP-GHO-1500 °C by utilizing the multiple peak fit options available in Origin Pro-2015 and using Lorentzian functions if all six peaks corresponded to the ordered pyrochlore. The OP Raman modes are shown in Figure 3b at 305, 384, 514, 518, 616, and 767cm^−1^. The Raman modes present at 767 cm^−1^ could be due to the distortion of the HfO_6_ octahedra [86].

In summary, the XRD and Raman results showed that our as-synthesized GHO-650 °C and GHO-1000 °C powders crystallized in the WO-GHO phase. With the increase in the grain size along with the crystalline domains, six distinct Rama peaks were visible for the GHO-1250 °C and GHO-1500 °C samples. The Raman spectra of the GHO-1250 °C and GHO-1500 °C samples (Figure 3) and the quantitative analysis (ESI-4) confirm the WO-to-OP phase evolution. The typical superlattice peaks in the XRD spectra are assumed to be absent due to the small volume of the diffraction domain (grain size) of the structure, in addition to the low resolving power of our diffractometer.

### 3.5. Magnetic Properties

**Figure 4:**
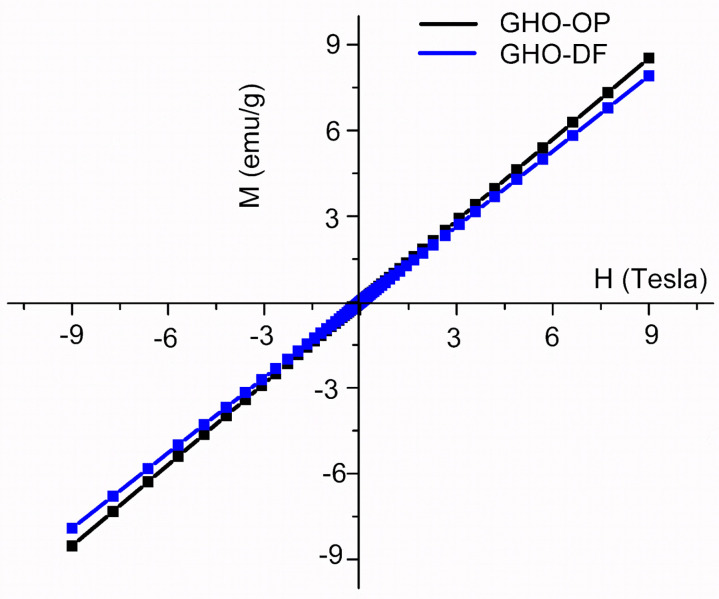
Magnetization curves for OP-GHO, compared with WO-GHO.

The magnetic measurements (M vs. H) on GHO-650 °C (WO-GHO) and GHO-1500 °C (OP-GHO) samples were carried out by using vibrating sample magnetometry at room temperature. Magnetization curves for the as-synthesized GHO-650 °C and GHO-1500 °C are presented in Figure 4. The linear (M vs. H) nature of the graph could be interpreted as both samples exhibiting either a weak ferromagnetic, antiferromagnetic, or paramagnetic behavior. However, this will be resolved via DFT calculations.

The maximum magnetization (M_max_) at ± 9 T was found to be 8.4% higher (8.5 emu/gm) for GHO-1500 °C (OP-GHO) relative to GHO-650 °C (WO-GHO), which was found to be 7.8 emu/gm. The higher value of M_max_ for OP-GHO compared to WO-GHO may be attributed to the enhanced periodicity of OP-GHO when compared to WO-GHO, induced by a higher thermal treatment. The magnetic properties in the GHO NCs originate at the Gd^3+^ ions, which have seven unpaired inner 4f electrons shielded by the crystal field of the outer closed-shell electrons in 5s^2^5p^6^. In addition to Gd^3+^ ions, other possible causes of the observed magnetic behavior in these samples could also be attributed to various other factors, such as F-centers, Hf^4+^ ion, cation vacancies, etc. [87]. We further performed electronic calculations in order to find the origin of the OP-GHO magnetic properties.

## 4. DFT Calculations in OP-GHO

### 4.1. Densities of States (DOS) Calculations

**Figure 5:**
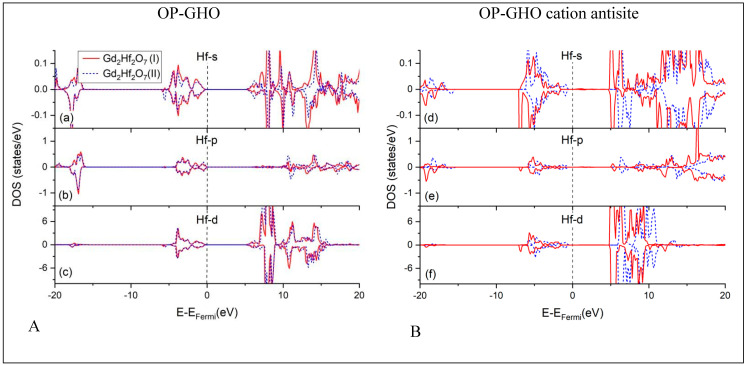
Hf DOS spectra per s, p, and d atomic orbitals for the OP-GHO structures, with the O_8a_sites simulated via ghost atoms (I) and being vacant (II). (**A**). (**a**)–(**c**) stable structures and (**B**). (**d**)–(**f**) cation antisites.

**Figure 6:**
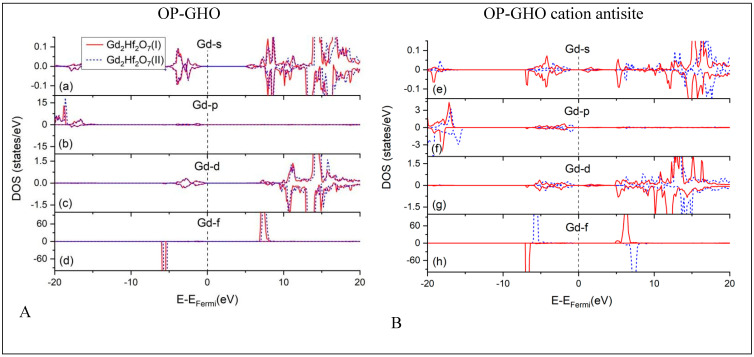
Gd DOS spectra per s, p, d, and f atomic orbitals for the OP-GHO structures, with the O_8a_ sites simulated via ghost atoms (I) and being vacant (II). (**A**). (**a**)–(**c**) stable structures and (**B**). (**d**)–(**f**) cation antisites.

**Figure 7:**
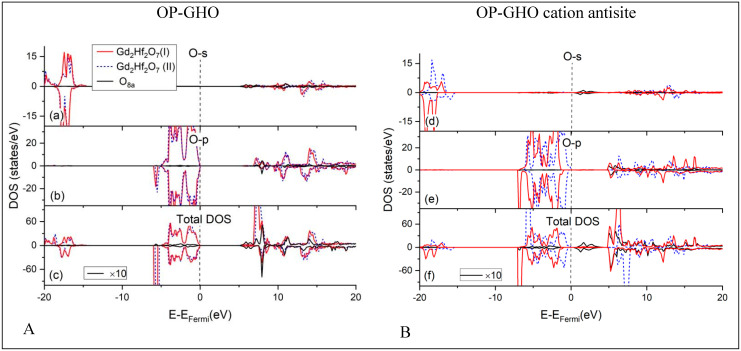
O DOS spectra per s and p atomic orbitals and total DOS for the OP-GHO structures, with the O_8a_ sites simulated via ghost atoms (I) and being vacant (II). (**A**). (**a**)–(**c**) stable structures and (**B**). (**d**)–(**f**) cation antisites.

Figure 5 and Figure 6 show the spin-resolved Hf and Gd DOS spectra per atomic orbital, respectively, for the structures of this work. Figure 7 shows the O DOS spectra per atomic orbital and the total DOS. Positive and negative DOS refer to spin-up and -down states, respectively. For the OP-GHO, its DOS spectra, produced with either the O_8a_ site being simulated via ghost atoms or remaining vacant, are almost identical. However, for the OP-GHO antisite, some shifts in the spectra between the two calculations are observed.

Magnetism is generally evidenced via spin-polarization around the Fermi energy area (Stoner magnetism) or by a concomitant shifting of the spin-up (spin-down) states above the Fermi energy and spin-down (spin-up) states below the Fermi energy (covalent magnetism) [88,89,90]. Covalent magnetism is found in both ferromagnetic and antiferromagnetic materials [88]. Gruber et al. stated that, in the covalent magnetism case, states became reweighted so that a spin-up case was doubly occupied whereas a spin-down was empty [91]. This effect is found in the Gd-4f states for the GHO and its cation antisite, both O_8a_ vacancy simulations (Figure 6d,h). Our observed shift of the Gd-4f states is in agreement with the computational work of Petersen et al. on Gd surfaces [45]. Both the GHO and its antisite are insulators. Additional calculations with the O_8a_ site vacancy show that the GHO and its antisite bandgaps are 5.24 eV and 5.96 eV, respectively. However, when the O_8a_ site is simulated with a ghost atom, the above values decrease slightly and appear at 5.04 eV and 5.77 eV, respectively. As expected, OP-GHO and its antisite are insulators.

DOS spectra could also be used to examine the chemical interaction via atomic orbital hybridizations and shifts in the atomic bands. In the energy region between –20 eV and –15 eV (relative to the Fermi energy), the bands of the GHO stable structures are dominated by the Gd-5p and O-2s orbitals (strong hybridization, Figure 6b and Figure 7a), in agreement with past calculations [74]. However, the Gd-5p orbital contributions for the GHO antisites at the above energy region are substantially decreased (Figure 6d), which decreases the hybridization between Gd-5p and O-2s orbitals for the cation antisites.

In the energy region between –6 eV and 0 eV (relative to Fermi energy), the bands are dominated by the O-2p orbitals, accompanied by smaller contributions from the Hf and Gd 5d orbitals, for all structures in this work (Figure 7): These effects lead to hybridization between the metals 5d orbitals and the O-2p orbitals, thus affecting the metal-O bonds. For all cases, the Gd-f orbitals appear away from the Fermi energy, and thus their contribution to chemistry changes is small.

Last, we examine the DOS in the energy region between 0 eV and 5 eV, the latter value coinciding with the bottom of the conduction band for the OP-GHO. Figure 7 reveals that the O_8a_ ghost contributions to the DOS spectra are due to O-2s orbitals and that these bands are hybrids of the O-2s with the metal bands.

### 4.2. Spin Density Calculations

**Figure 8:**
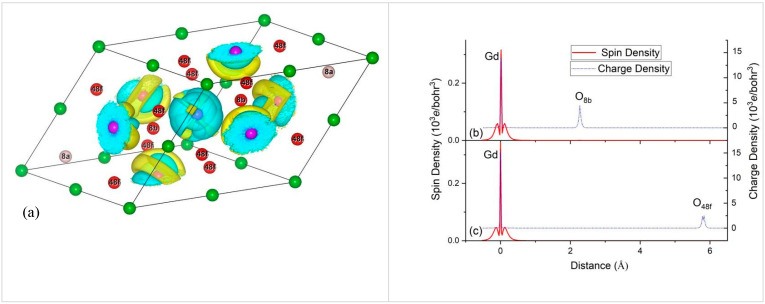
(**a**) The OP-GHO spin density isosurfaces. Isodensity surfaces of 0.0015 *e*/bohr^3^ are displayed. Lines show the unit cell boundaries. Colors are atoms (Green = Hf; Purple = Gd; Red = O; Pink = O ghost atom). The O sites are shown. Yellow and cyan represent spin-up and -down cases, respectively. (**b**,**c**) represent the spin and charge density profiles. Charge density peaks denote nuclear positions.

**Figure 9:**
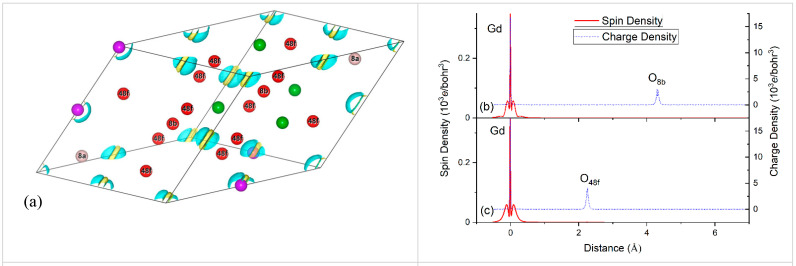
(**a**) OP-GHO antisite spin density isosurfaces. Isodensity surfaces of 0.0015 *e*/bohr^3^ are displayed. Lines show the unit cell boundaries. Colors are atoms (Green = Hf; Purple = Gd; Red = O; Pink = O ghost atom). The O sites are shown. Yellow and cyan represent spin-up and -down cases, respectively. (**b**,**c**) represent the spin and charge density profiles. Charge density peaks denote nuclear positions.

The origin of the magnetism is also determined via spin density calculations. Figure 8 and Figure 9 show the spin density isodensity surfaces and profiles of the OP-GHO and its antisite, with the O_8a_ sites simulated via ghost atoms. For both cases, a nonzero spin density is evident only at the Gd sites and is absent at the O and Hf sites. For the OP-GHO and its antisite, the Gd spin density profiles show spin density maxima at the Gd nuclear positions. Additional Mulliken population calculations show that the spin density (i.e., |$e_{spin↑}-e_{spin\;↓}|$) of the OP-GHO cation and its antisite is only due to the Gd atom.

We calculated the energy of the OP-GHO antiferromagnetic configuration using a supercell double the size (unit cell 44 atoms) of its corresponding ferromagnetic case (unit cell 22 atoms). The energy difference between the energies of the antiferromagnetic and the doubled energy of the ferromagnetic configuration is −1.5 × 10^−4^ Hartree. This value is lower than the expected error of 10^−6^ Hartree between supercell geometries, and, thus, the above value is reliable. Therefore, OP-GHO is antiferromagnetic. However, the exact opposite was observed for the GHO antisite. Using the 44 atom supercell, we found that the ferromagnetic site was the preferred configuration for the GHO antisite.

## 5. Overlap Populations COOP and COHP

Table 2 shows the COHP and COOP values for the Hf-Gd and the metal -O_48f, 8b_ (metal = Hf, Gd) for the OP-GHO and its cation antisite. Recall that negative -COHP and COOP are indicative of covalent destabilizing (i.e., antibonding) interactions, whereas small COHP and COOP values are indicative of nonbonding interactions. All of the above interactions are either small destabilizing or nonbonding, for both the GHO and its antisite (Table 2). For the GHO, the COHP shows that the Hf-O_48f_ and the Gd-O_8d_ are the most destabilizing interactions, whereas for the antisite three destabilizing interactions are of importance: the Gd-O_48f_ and the Hf-O_48f, 8b_. These results are in contrast with the past report of Li et al. on GHO, where they reported positive COOP for the OP-GHO metal-O_48f_ (i.e., bonding interaction) [74]. Although Li et al. stated that GHO stability is due to ionic bonding, their positive COOP values enhanced the ionic bonding, whereas the opposite was observed here. This disagreement could be attributed to the different methods employed. Therefore, the presence of small covalent metal-O destabilizing interactions shows that metal-O bonds are formed due to ionic interactions, which more than offset these small covalent destabilizing interactions (whenever present). Table S2 shows that the O_8a_ vacancy site of the GHO, when simulated via a ghost atom, has a small negative gross population of –0.34 *e*. This negative value is not an artifact of the calculations but is indicative of an anomalous antibonding mixing of the involved orbitals [92] (counterintuitive orbital mixing between O_8a_ 3s and 3p). Therefore, the Mulliken gross atom populations show that the vacancy site is charged when simulated via ghost atoms, which is missed otherwise. This observation shows an advantage of mimicking the O_8a_ vacancy site via ghost atoms relative to leaving this site vacant.

## 6. Conclusions

This work highlights combined experimental and computational approaches to elucidate the electronic structure, bonding, and magnetic properties of Gd_2_Hf_2_O_7_ (GHO). SEM results suggested the formation of nanoparticles after coprecipitation, followed by a molten salt treatment in GHO. A structural evolution analysis using XRD and Raman spectroscopy suggested a weakly order (WO)-order pyrochlore (OP) phase evolution in GHO. A low temperature favoring WO and a thermal treatment beyond 1250 °C favored a full OP phase. SQUID magnetic measurements suggested a weak magnetism for both WO-GHO and OP-GHO. DFT calculations showed that the GHO magnetism and its antisite were due to the shift in the localized Gd-4f states above and below the Fermi level. The OP-GHO was antiferromagnetic, whereas its antisite was ferromagnetic, and both substances were insulators. The localization of the Gd-4f states was indicative of weak magnetism. Our DFT calculations simulated the oxygen defect site using two distinct approaches. Ghost atom simulations showed a small charge build-up at the defect site. Overlap population calculations evidenced small destabilizing interactions for the metal-oxygens of both the OP-GTO and its antisite. This observation is in contrast with past reports and could be attributed to the basis set’s dependency on these calculations. The metal-oxygen interactions can only be stabilized via ionic interactions. Thus, the metal-oxygen ionic bonds play an important role in stabilizing the defect structure.

## Figures and Tables

**Table 1:** Lattice parameters (a and x) and the nearest metal-metal and metal-O interatomic distances for the DFT-optimized OP-GHO structures. Values in parentheses refer to the cation antisites.

OP-GHO	Lattice Parameters		Distances				
	a	*x*	Hf-Gd	Gd-O_8b_	Gd-O_48f_	Hf-O_8b_	Hf-O_48f_
	**(Å)**						
O_8a_ ghost atoms calc.	10.473	0.3368	3.702	2.267	2.519	4.342	2.063
	(10.427)	(0.3767)	(3.687)	(4.323)	(2.267)	(2.258)	(2.248)
O_8a_ vacant calc.	10.397	0.3377	3.676	2.251	2.495	4.310	2.052
	(10.254)	(0.3751)	(3.625)	(4.261)	(2.227)	(2.226)	(2.224)
Other calc. [79]	10.55	0.337					
[74]	10.53	0.3367		2.2798	2.5339		2.0734
Experimental [78]	10.49						

**Table 2:** COHP and COOP values for the OP-GHO Hf-Gd and metal-O_48f, 8b_ (metal = Hf, Gd) interactions. Values in parentheses are for the antisite. The -COHP, COOP < 0 are indicative of destabilizing interactions.

Atom Pair	GHO	
	O_8a_ via Ghost Atoms	O_8a_vacant
	-COHP	
Hf-Gd	–0.002 (–0.016)	<0.001 (−0.014)
Gd-O_8b_	−0.086 (<0.001)	−0.088 (−0.006)
Gd-O_48f_	−0.023 (−0.062)	−0.024 (−0.170)
Hf-O_8b_	<0.001 (−0.027)	<0.001 (−0.036)
Hf-O_48f_	−0.107 (−0.031)	−0.111 (−0.068)
	COOP	
Hf-Gd	< 0.001 (<0.001)	<0.001 (−0.009)
Gd-O_8b_	−0.042 (<0.001)	−0.043 (−0.033)
Gd-O_48f_	−0.010 (−0.027)	−0.011 (−0.103)
Hf-O_8b_	<0.001 (0.020)	<0.001 (−0.048)
Hf-O_48f_	−0.041 (0.012)	−0.045 (−0.067)

## Supplementary Materials

The following are available online. SEM images of the as-synthesized Gd_2_Hf_2_O_7_ nanocrystals and samples calcined at 1000 °C, 1250 °C, and 1500 °C for six hours in the air, samples of CRYSTAL17 input files, Mulliken gross atom populations for OP-GHO, additional information on computational methodology, FWHM, Scherrer calculations of their particle sizes, and the Raman spectra analysis can be found in the supplementary file.
