# Supplementary material for: Structural Evolution and Magnetic Properties of Gd2Hf2O7 Nanocrystals: Computational and Experimental Investigations
Source: Molecules. 2020 Oct 21;25(20):4847. doi: 10.3390/molecules25204847 (PMC7587975; doi:10.3390/molecules25204847)
Supplement: Supplementary file 1 [file molecules-25-04847-s001.pdf]

Supplementary Material

Structural Evolution and Magnetic Properties of $Gd_2Hf_2O_7$ Nanocrystals: Computational and Experimental Investigations

Madhab Pokhrel[1*], Nicholas Dimakis[1*], Chamath Dannangoda[1], Santosh K. Gupta[2,], Karen S. Martirosyan[1], and Yuanbing Mao[3*]

[1]Department of Physics and Astronomy, University of Texas Rio Grande Valley, 1201 W University Drive, Edinburg, Texas 78539, USA

[2]Radiochemistry Division, Bhabha Atomic Research Centre, Trombay, Mumbai-400085, India

[3]Department of Chemistry, Illinois Institute of Technology, 3105 South Dearborn Street, Chicago, IL 60616, USA

Correspondence: madhab.pokhrel@utrgv.edu (M. P.); nicholas.dimakis@utrgv.edu (N. D.); ymao17@iit.edu (Y. M.); Tel: +1-956-665-8761 (N. D.); 956-665-7360 (M. P.); Tel:+1-312-567-3815 (Y. M.)

ESI-1: The SEM images of the as prepared $Gd_2Hf_2O_7$ (GHO) powder at 650 °C and samples calcinated to 1000 °C, 1250 °C and 1500 °C for 6 hrs. in air.

$Gd_2Hf_2O_7$ powder sample was prepared via the co-precipitation method, followed by a molten-salt method. The synthesis routes have been extensively reported previously[1]. The as-prepared GHO powder was calcined at 1000 °C, 1250 °C, and 1500 °C temperatures in air.

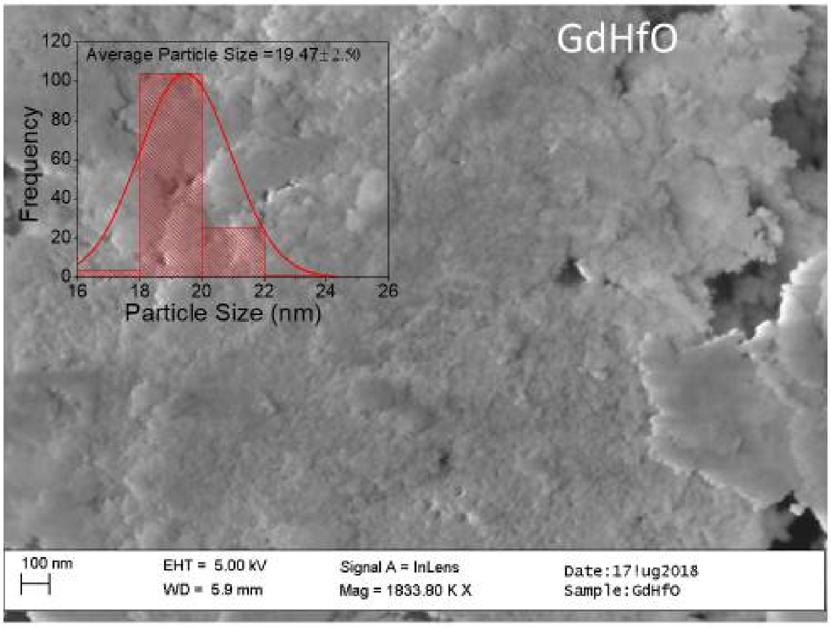

Fig. ESI-1a shows the SEM images of the as synthesized nanocrystals at 650 °C.

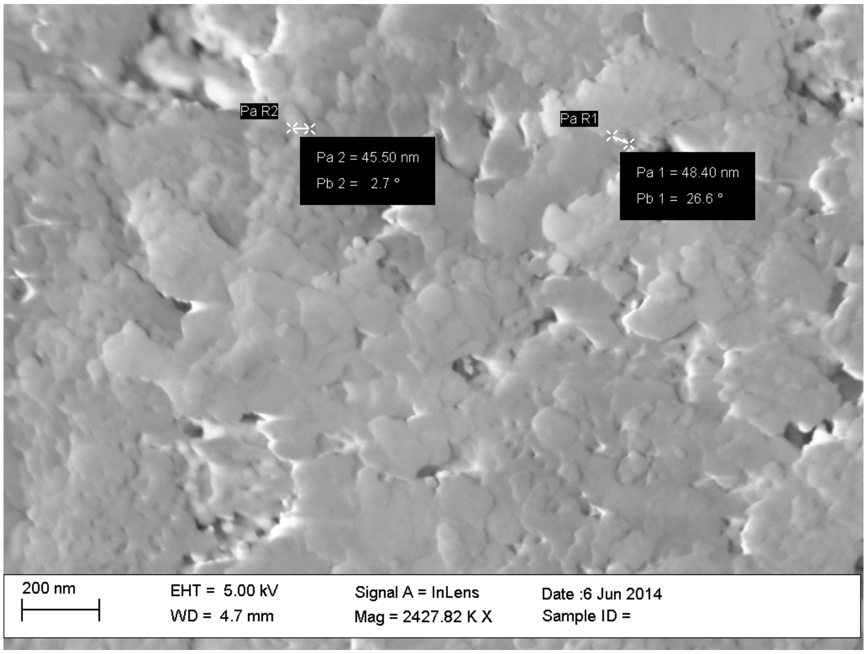

Fig. ESI-1b shows the SEM images of the as particles calcined to 1000 ℃.

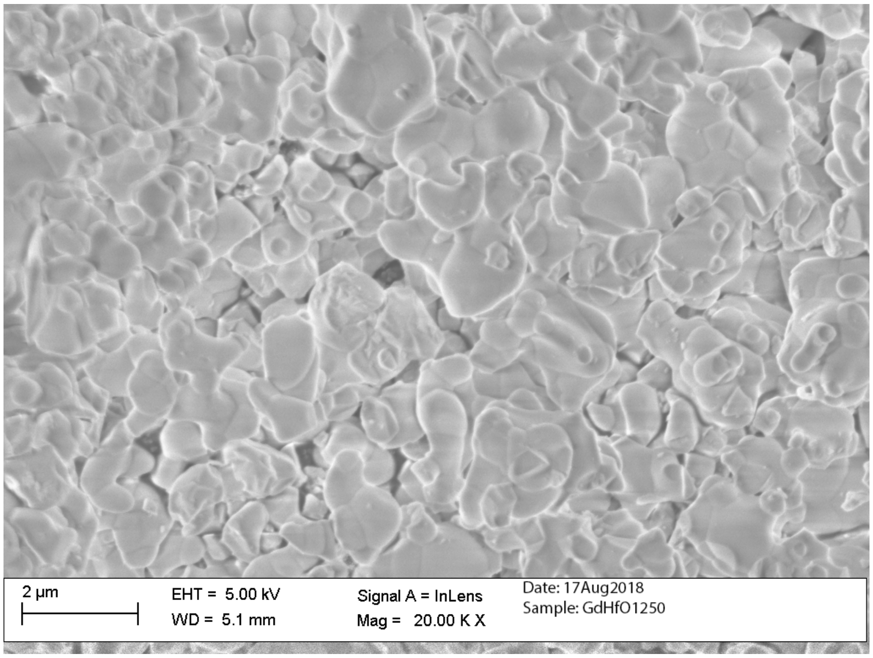

Fig. ESI-1c shows the SEM images of the as particles calcined to 1250 ℃.

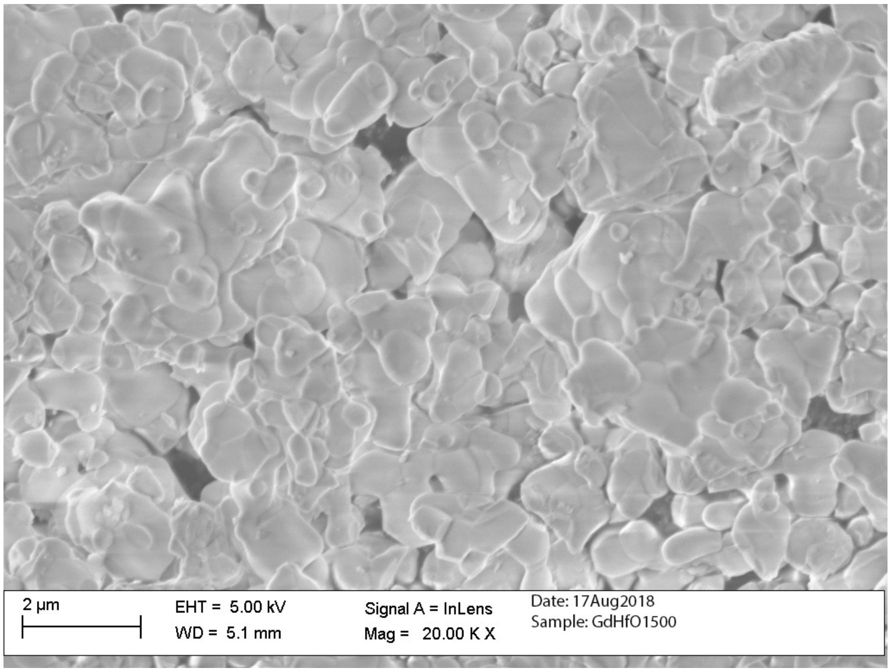

Fig. ESI-1d shows the SEM images of the as particles calcined to 1500 ℃.

ESI-2: Sample Characterization

X-ray diffraction (XRD) was utilized to evaluate the crystalline phase of as synthesized and calcinated compounds under different temperatures. XRD patterns were collected using the Bruker D8 ADVANCE x-ray diffractometer with Cu Kα_1 radiation (λ = 0.15406 nm). Raman spectra were collected using the Bruker Senterra-system using a 785 nm helium-neon laser with a spatial resolution of 2 μm.

ESI-3. Calculation of lattice parameters and Scherrer evaluation of particle size for $Gd_2Hf_2O_7$ from XRD patterns

Profile fitting is a precise method to determine diffraction peak position, intensity, and full width half maximum (FWHM) for calculating lattice parameters and crystallite size. Cubic crystal lattice parameter are calculated using the equation:

$$a = d\sqrt{h^2 + k^2 + l^2}$$

where d can be calculated from the position of the peak and (hkl) correspond to the miller index. The Sherrer equation gives an estimate of the size of sub-micrometer particles. It can be calculated using the following equation:

$$\tau = \frac{K\lambda}{\beta \cos\theta}$$

where τ is the mean size of the crystalline domains, K is a shape factor (in this case given a value of 0.94), λ is the wavelength (1.5406 Å), β is the line broadening at the FWHM, and θ is the Bragg angle.

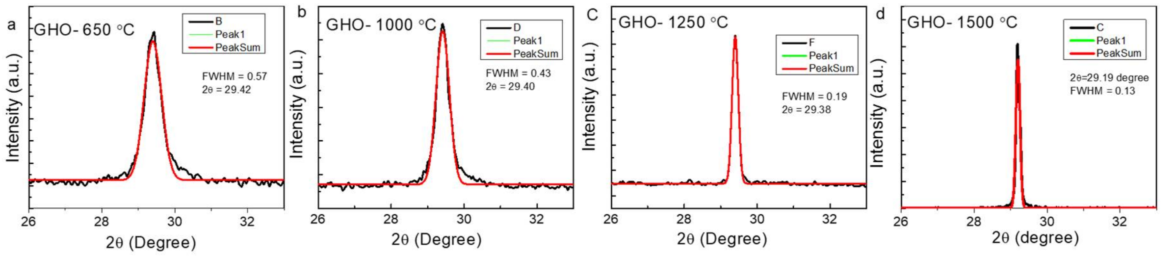

Figure S1. Gaussian fitting to (222) peak assuming that (a) $Gd_2Hf_2O_7$-650 °C, (b) $Gd_2Hf_2O_7$-1000 °C are crystallized in weakly ordered pyrochlore phase. In addition, assuming that (c) $Gd_2Hf_2O_7$-1250 °C, (d) $Gd_2Hf_2O_7$-1500 °C are crystallized in fully ordered pyrochlore phase, Gaussian fitting to (222) was evaluated. The values used for the calculation of lattice parameters and particle sizes for all samples have been listed in Table S1.

Table S1. The lattice parameters and Scherrer evaluation of the crystalline size of the $Gd_2Hf_2O_7$ Powder

$Gd_2Hf_2O_7$	**2θ (degrees)**	**θ (radians)**	**Lattice con. *a* (Å)**	**FWHM (degrees)**	**FWHM β(radians) instrumental corrections**	**Particle size τ (nm)**

**650**	29.19	0.254	10.49	0.573	0.009	≈ 18 nm
**1000**	29.19	0.254	10.49	0.457	0.0079	*≈42 nm*
**1250**	29.18	0.255	10.50	0.195	0.0035	
**1500**	29.18	0.255	10.50	0.137	0.0024	

Scherrer evaluation of the crystalline size is reliable if the crystalline size is smaller than 100 nm, so we have limited our calculations for $Gd_2Hf_2O_7$-650 °C, and for $Gd_2Hf_2O_7$-1000 °C.

ESI-4. Raman Spectra: Fitted Raman spectra of the GHO nanocrystals annealed at 650 °C and 1500 °C for 6 hrs in a box furnace. Quantitative analysis of the full width at half maximum (FWHM) cleary shows difference between the Raman spectra at 650 ºC and 1500 ºC data. For as-synthesized GHO-650 ºC powder, two distinct broad peaks centered at 305 cm^{-1} with FWHM of 53 cm^{-1} and 406 cm^{-1} with FWHM of 163 cm^{-1} were observed. But the fitted Raman modes for OP-GHO-1500 ºC shows all the six peaks as expected for fully ordered pyrochlore GHO. The difference in FWHM especially for GHO-1500 sample corresponding to peaks at 305 (FWHM =47 cm^{-1}), 384 ((FWHM =43 cm^{-1}) compared to those of GHO-650 ºC shows the clear difference between these two proposed phases. It particular, new peaks were observed on Raman spectra for GHO-1500 ºC samples and existing peaks were sharpened and shifted in comparsion to the GHO-650 ºC.

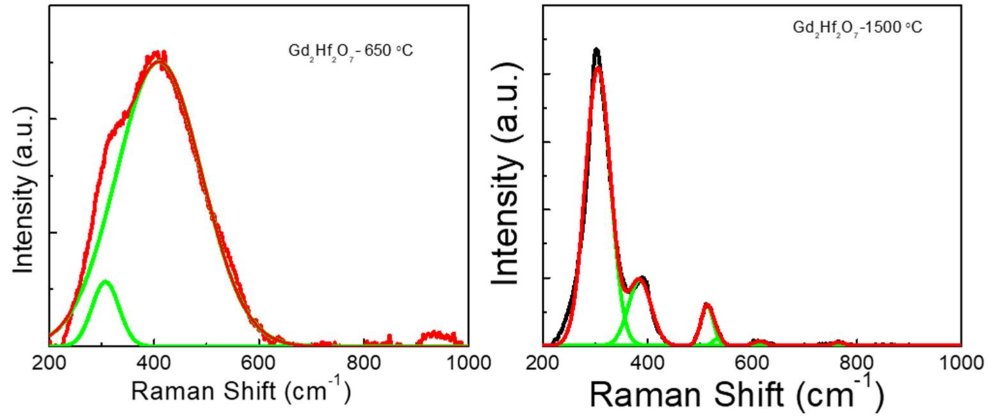

Figure S2. Gaussian fitted Raman spectra of the GHO nanocrystals annealed at 650 °C and 1500 °C for 6 hrs in a box furnace

ESI-5. CRYSTAL17 input files

a) *d12 input file for the OP-GHO with O_{8a} site being vacant.

```
pyrochlore
EXTERNAL
OPTGEOM
FULLOPTG
FINALRUN
4
END
END
264 10
INPUT
36. 0 1 1 1 1 1
 24.602151 637.200869 0
 16.889250 261.689601 0
 13.643358 106.856533 0
 24.126917 -50.683590 0
 22.131887 -27.579630 0
0 1 5 8.0 1.0
 16558.82178440 0.000645997107804 -1.12000911838e-05
 8281.241814270 0.000456737868268 -1.15443727285e-05
 2428.055307600 0.004957200977550 -0.000279039727156
 686.9343502650 0.014754520214900 -0.001770821412680
 203.3273442580 0.023272000630700 -0.011218549383300
```

```
0 1 4 8.0 1.0
 58.59312787820 -0.04358445990370 0.0332206857579000
 31.37450772520 0.307726523258000 0.1134727368690000
 14.66781071180 -0.81384023087300 -0.753153463181000
 5.154353221900 -0.14585294630900 -0.102952329736000
0 1 3 2.0 1.0
 7.324626990620 -0.02628572881510 0.1309704451850000
 4.750191474880 0.867947304477000 0.4353834497150000
 2.394851856570 0.620814739348000 0.3782597584940000
0 1 2 0.0 1.0
 1.748069516900 -0.18922264531600 0.1926673942280000
 0.724554798716 1.442760719820000 0.9051984070640000
0 1 1 0.0 1.0
 0.291297374783 1.000000000000000 1.0000000000000000
0 3 7 10.0 1.0
 241.8557695170 0.001664378861840
 95.38901905500 0.007934096199710
 37.31028452020 0.033360200154700
 11.46968024070 0.109631922757000
 6.120347442000 0.420903140707000
 3.356448252970 0.363928472434000
 2.080067882430 0.243434996082000
0 3 1 1.0 1.0
 1.007109020984 1.000000000000000
0 4 4 7.0 1.0
 92.08046827710 0.007549697051550
 33.86221461070 0.060555558750800
 14.28080916500 0.168094391859000
 6.210178574470 0.266032200486000
0 4 3 0.0 1.0
 2.908308038960 0.403786353963000
 1.554949827380 0.288405917751000
 0.770540575731 0.215866476748000
0 4 1 0.0 1.0
 0.374168528890 1.000000000000000
272 5
INPUT
12. 1 3 3 2 0 0
    1.14090     -2.59020 -1
   18.98210      5.08602 -2
    4.32002    154.12616  0
    3.92413    -76.34646  0
    1.11008      3.80263 -2
    2.58171    159.39488  0
    2.38920   -133.12772  0
   21.00000      5.23486 -2
```

```
   2.80487     30.09851  0
0 1 4 8.0 1.0
   4.711    0.403273   0.013791
   3.331   -0.996540  -0.179991
   0.9844   0.979338   0.693601
   0.3587   0.445791   0.461877
0 1 1 2.0 1.0
   1.13         1.0        1.0
0 1 1 0.0 1.0
   0.215        1.0        1.0
0 3 3 2.0 1.0
    3.1991     -0.005062
    0.9007      0.427636
    0.3303      0.667943
0 3 1 0.0 1.0
   0.20     1.0
239 11
INPUT
11. 0 2 4 4 2 0
  7.858275 135.134974 0
  3.382128  15.411632 0
  6.849791  29.251437 0
  6.710092  58.508363 0
  3.042159   3.780243 0
  2.937330   7.676547 0
  5.416315  11.849911 0
  5.333416  17.778103 0
  1.976212   2.062383 0
  1.961111   3.075654 0
  5.028590  -6.928078 0
  5.005582  -9.155099 0
0 0 2 2 1.0
  10.0000000000     -0.174876867960
  8.50000000000      0.341523450840
0 0 1 0 1.0
  3.36539055000      1.000000000000
0 0 1 0 1.0
  0.68860679000      1.000000000000
0 0 1 0 1.0
  0.31191503000      1.000000000000
0 2 4 6 1.0
  8.05540936160      0.036410978939
  4.01634587510     -0.208725725470
  1.02329154040      0.489569293350
  0.49843561636      0.606119438330
0 2 1 0 1.0
```

0.53307662000 1.000000000000
0 2 1 0 1.0
0.18700948000 1.000000000000
0 3 3 3 1.0
3.90308330600 -0.009820979262
1.13632343020 0.191836296640
0.45617043877 0.405977885390
0 3 1 0 1.0
0.33346286000 1.000000000000
0 3 1 0 1.0
0.18197880000 1.000000000000
0 4 1 0 1.0
0.26515000000 1.000000000000
6 8
0 0 6 2.0 1.0
13575.349682 0.00022245814352
2035.2333680 0.00172327382520
463.22562359 0.00892557153140
131.20019598 0.03572798450200
42.853015891 0.11076259931000
15.584185766 0.24295627626000
0 0 2 2.0 1.0
6.2067138508 0.41440263448000
2.5764896527 0.23744968655000
0 0 1 0.0 1.0
0.4941102000 1.00000000000000
0 0 1 0.0 1.0
0.1644071000 1.00000000000000
0 2 4 2.0 1.0
34.697232244 0.00533336578050
7.9582622826 0.03586410909200
2.3780826883 0.14215873329000
0.8143320818 0.34270471845000
0 2 1 0.0 1.0
0.5662417100 1.00000000000000
0 2 1 0.0 1.0
0.2673545000 1.00000000000000
0 3 1 0.0 1.0
0.8791584200 1.00000000000000
257 4
HAYWSC
0 1 4 8.0 1.0
14.683106378 -0.002299267 -0.001255610
4.555053782 0.042230707 0.001376938
1.309599879 0.054322099 -0.326200098
1.292413209 -0.80 0.25

```
 0 1 1  2.0 1.0
0.668647836    1.0    1.0
 0 1 1  0.0 1.0
0.254637078    1.0    1.0
 0 3 1  1.0 1.0
0.363426121     1.0
242 8
INPUT
14. 0 2 4 4 2 0
  10.097000 180.076853 0
   4.375670  24.715920 0
   9.126564  41.227678 0
   8.863223  82.452670 0
   4.044948   6.345092 0
   3.866657  12.458423 0
   7.535754  19.308744 0
   7.278976  28.977674 0
   2.763205   3.189516 0
   2.772085   4.700169 0
   6.306633  -7.178888 0
   6.356448  -9.745978 0
0 0 3 2 1.0
  7.20338000000     -0.893106172370
  5.05229500000      1.607409575600
  2.91353300000      0.278041860880
0 0 1 2 1.0
  0.87390909000      1.000000000000
0 0 1 0 1.0
  0.42606630000      1.000000000000
0 2 4 6 1.0
  3.15186600000     -0.718730408720
  2.45348200000      0.764428878700
  0.85030192180      0.648085856400
  0.36918237179      0.267921152340
0 2 1 0 1.0
  0.48549962000      1.000000000000
0 3 4 4 1.0
  5.00444454970     -0.021587364862
  1.77368233240      0.209586800860
  0.76950591696      0.437308805990
  0.31530878939      0.411239270090
0 3 1 0 1.0
  0.34752659000      1.000000000000
0 4 1 0 1.0
  1.05545000000      1.000000000000
8 7
```

```
0 0 8 2. 1.
 8020.  0.00108
 1338.  0.00804
 255.4  0.05324
 69.22  0.1681
 23.90  0.3581
 9.264  0.3855
 3.851  0.1468
 1.212  0.0728
0 1 4 6. 1.
 49.43 -0.011 0.0097
 10.47 -0.091 0.069
 3.235 -0.039 0.207
 1.22 0.379 0.347
0 1 1 0. 1.
 0.459019090935  1. 1.
0 1 1 0. 1.
 0.166171528548 1. 1.
0 3 1 0. 1.
 0.883705090207  1.
0 3 1 0. 1.
 0.296566440786  1.
0 4 1 0. 1.
 0.602777853728  1.
99 0
END
DFT
HSE06-D3
SPIN
XLGRID
END
SCFDIR
REPLDATA
HIRSHBLK
XLGRID
ITCONV
8
MXNUMITER
100
END
TOLINTEG
8 8 8 8 16
SHRINK
12 12
SMEAR
0.005
```

FMIXING
90
ANDERSON
MAXCYCLE
1500
TOLDEE
9
DIIS
END

b) External *gui file used for a)

```
 3   5   6 E -4.3125015776157E+03 DE 2.8E-10(114)
 0.000000000000E+00  0.519835104004E+01  0.519835104004E+01
 0.519835104004E+01  0.000000000000E+00  0.519835104004E+01
 0.519835104004E+01  0.519835104004E+01  0.000000000000E+00
  48
 0.100000000000E+01  0.000000000000E+00  0.000000000000E+00
 0.000000000000E+00  0.100000000000E+01  0.000000000000E+00
 0.000000000000E+00  0.000000000000E+00  0.100000000000E+01
 0.000000000000E+00  0.000000000000E+00  0.000000000000E+00
-0.100000000000E+01  0.000000000000E+00  0.000000000000E+00
 0.000000000000E+00 -0.100000000000E+01  0.000000000000E+00
 0.000000000000E+00  0.000000000000E+00  0.100000000000E+01
 0.259917552002E+01  0.259917552002E+01  0.000000000000E+00
 0.100000000000E+01  0.000000000000E+00  0.000000000000E+00
 0.000000000000E+00 -0.100000000000E+01  0.000000000000E+00
 0.000000000000E+00  0.000000000000E+00 -0.100000000000E+01
 0.000000000000E+00  0.259917552002E+01  0.259917552002E+01
-0.100000000000E+01  0.000000000000E+00  0.000000000000E+00
 0.000000000000E+00  0.100000000000E+01  0.000000000000E+00
 0.000000000000E+00  0.000000000000E+00 -0.100000000000E+01
 0.259917552002E+01  0.000000000000E+00  0.259917552002E+01
 0.000000000000E+00  0.000000000000E+00  0.100000000000E+01
 0.100000000000E+01  0.000000000000E+00  0.000000000000E+00
 0.000000000000E+00  0.100000000000E+01  0.000000000000E+00
 0.000000000000E+00  0.000000000000E+00  0.000000000000E+00
 0.000000000000E+00  0.100000000000E+01  0.000000000000E+00
 0.000000000000E+00  0.000000000000E+00  0.100000000000E+01
 0.100000000000E+01  0.000000000000E+00  0.000000000000E+00
 0.000000000000E+00  0.000000000000E+00  0.000000000000E+00
 0.000000000000E+00  0.000000000000E+00 -0.100000000000E+01
-0.100000000000E+01  0.000000000000E+00  0.000000000000E+00
 0.000000000000E+00  0.100000000000E+01  0.000000000000E+00
 0.259917552002E+01  0.259917552002E+01  0.000000000000E+00
```

0.000000000000E+00	-0.100000000000E+01	0.000000000000E+00
0.000000000000E+00	0.000000000000E+00	0.100000000000E+01
-0.100000000000E+01	0.000000000000E+00	0.000000000000E+00
0.259917552002E+01	0.000000000000E+00	0.259917552002E+01
0.000000000000E+00	0.000000000000E+00	0.100000000000E+01
-0.100000000000E+01	0.000000000000E+00	0.000000000000E+00
0.000000000000E+00	-0.100000000000E+01	0.000000000000E+00
0.000000000000E+00	0.259917552002E+01	0.259917552002E+01
0.000000000000E+00	-0.100000000000E+01	0.000000000000E+00
0.000000000000E+00	0.000000000000E+00	-0.100000000000E+01
0.100000000000E+01	0.000000000000E+00	0.000000000000E+00
0.259917552002E+01	0.259917552002E+01	0.000000000000E+00
0.000000000000E+00	0.000000000000E+00	-0.100000000000E+01
0.100000000000E+01	0.000000000000E+00	0.000000000000E+00
0.000000000000E+00	-0.100000000000E+01	0.000000000000E+00
0.259917552002E+01	0.000000000000E+00	0.259917552002E+01
0.000000000000E+00	0.100000000000E+01	0.000000000000E+00
0.000000000000E+00	0.000000000000E+00	-0.100000000000E+01
-0.100000000000E+01	0.000000000000E+00	0.000000000000E+00
0.000000000000E+00	0.259917552002E+01	0.259917552002E+01
0.000000000000E+00	-0.100000000000E+01	0.000000000000E+00
-0.100000000000E+01	0.000000000000E+00	0.000000000000E+00
0.000000000000E+00	0.000000000000E+00	-0.100000000000E+01
0.000000000000E+00	0.000000000000E+00	0.000000000000E+00
0.000000000000E+00	0.100000000000E+01	0.000000000000E+00
0.100000000000E+01	0.000000000000E+00	0.000000000000E+00
0.000000000000E+00	0.000000000000E+00	-0.100000000000E+01
0.259917552002E+01	0.259917552002E+01	0.000000000000E+00
0.000000000000E+00	0.100000000000E+01	0.000000000000E+00
-0.100000000000E+01	0.000000000000E+00	0.000000000000E+00
0.000000000000E+00	0.000000000000E+00	0.100000000000E+01
0.259917552002E+01	0.000000000000E+00	0.259917552002E+01
0.000000000000E+00	-0.100000000000E+01	0.000000000000E+00
0.100000000000E+01	0.000000000000E+00	0.000000000000E+00
0.000000000000E+00	0.000000000000E+00	0.100000000000E+01
0.000000000000E+00	0.259917552002E+01	0.259917552002E+01
-0.100000000000E+01	0.000000000000E+00	0.000000000000E+00
0.000000000000E+00	0.000000000000E+00	-0.100000000000E+01
0.000000000000E+00	-0.100000000000E+01	0.000000000000E+00
0.000000000000E+00	0.000000000000E+00	0.000000000000E+00
0.000000000000E+00	0.000000000000E+00	-0.100000000000E+01
0.000000000000E+00	-0.100000000000E+01	0.000000000000E+00
-0.100000000000E+01	0.000000000000E+00	0.000000000000E+00
0.000000000000E+00	0.000000000000E+00	0.000000000000E+00
0.100000000000E+01	0.000000000000E+00	0.000000000000E+00
0.000000000000E+00	0.000000000000E+00	0.100000000000E+01

0.000000000000E+00	-0.100000000000E+01	0.000000000000E+00
0.259917552002E+01	0.259917552002E+01	0.000000000000E+00
0.100000000000E+01	0.000000000000E+00	0.000000000000E+00
0.000000000000E+00	0.000000000000E+00	-0.100000000000E+01
0.000000000000E+00	0.100000000000E+01	0.000000000000E+00
0.259917552002E+01	0.000000000000E+00	0.259917552002E+01
0.000000000000E+00	0.000000000000E+00	-0.100000000000E+01
0.000000000000E+00	0.100000000000E+01	0.000000000000E+00
0.100000000000E+01	0.000000000000E+00	0.000000000000E+00
0.000000000000E+00	0.259917552002E+01	0.259917552002E+01
0.000000000000E+00	0.000000000000E+00	0.100000000000E+01
0.000000000000E+00	0.100000000000E+01	0.000000000000E+00
-0.100000000000E+01	0.000000000000E+00	0.000000000000E+00
0.259917552002E+01	0.259917552002E+01	0.000000000000E+00
-0.100000000000E+01	0.000000000000E+00	0.000000000000E+00
0.000000000000E+00	0.000000000000E+00	0.100000000000E+01
0.000000000000E+00	0.100000000000E+01	0.000000000000E+00
0.000000000000E+00	0.259917552002E+01	0.259917552002E+01
0.000000000000E+00	0.000000000000E+00	0.100000000000E+01
0.000000000000E+00	-0.100000000000E+01	0.000000000000E+00
0.100000000000E+01	0.000000000000E+00	0.000000000000E+00
0.259917552002E+01	0.000000000000E+00	0.259917552002E+01
-0.100000000000E+01	0.000000000000E+00	0.000000000000E+00
0.000000000000E+00	-0.100000000000E+01	0.000000000000E+00
0.000000000000E+00	0.000000000000E+00	-0.100000000000E+01
0.000000000000E+00	0.000000000000E+00	0.000000000000E+00
0.100000000000E+01	0.000000000000E+00	0.000000000000E+00
0.000000000000E+00	0.100000000000E+01	0.000000000000E+00
0.000000000000E+00	0.000000000000E+00	-0.100000000000E+01
0.259917552002E+01	0.259917552002E+01	0.000000000000E+00
-0.100000000000E+01	0.000000000000E+00	0.000000000000E+00
0.000000000000E+00	0.100000000000E+01	0.000000000000E+00
0.000000000000E+00	0.000000000000E+00	0.100000000000E+01
0.000000000000E+00	0.259917552002E+01	0.259917552002E+01
0.100000000000E+01	0.000000000000E+00	0.000000000000E+00
0.000000000000E+00	-0.100000000000E+01	0.000000000000E+00
0.000000000000E+00	0.000000000000E+00	0.100000000000E+01
0.259917552002E+01	0.000000000000E+00	0.259917552002E+01
0.000000000000E+00	0.000000000000E+00	-0.100000000000E+01
-0.100000000000E+01	0.000000000000E+00	0.000000000000E+00
0.000000000000E+00	-0.100000000000E+01	0.000000000000E+00
0.000000000000E+00	0.000000000000E+00	0.000000000000E+00
0.000000000000E+00	-0.100000000000E+01	0.000000000000E+00
0.000000000000E+00	0.000000000000E+00	-0.100000000000E+01
-0.100000000000E+01	0.000000000000E+00	0.000000000000E+00
0.000000000000E+00	0.000000000000E+00	0.000000000000E+00

0.000000000000E+00	0.000000000000E+00	0.100000000000E+01
0.100000000000E+01	0.000000000000E+00	0.000000000000E+00
0.000000000000E+00	-0.100000000000E+01	0.000000000000E+00
0.259917552002E+01	0.259917552002E+01	0.000000000000E+00
0.000000000000E+00	0.100000000000E+01	0.000000000000E+00
0.000000000000E+00	0.000000000000E+00	-0.100000000000E+01
0.100000000000E+01	0.000000000000E+00	0.000000000000E+00
0.259917552002E+01	0.000000000000E+00	0.259917552002E+01
0.000000000000E+00	0.000000000000E+00	-0.100000000000E+01
0.100000000000E+01	0.000000000000E+00	0.000000000000E+00
0.000000000000E+00	0.100000000000E+01	0.000000000000E+00
0.000000000000E+00	0.259917552002E+01	0.259917552002E+01
0.000000000000E+00	0.100000000000E+01	0.000000000000E+00
0.000000000000E+00	0.000000000000E+00	0.100000000000E+01
-0.100000000000E+01	0.000000000000E+00	0.000000000000E+00
0.259917552002E+01	0.259917552002E+01	0.000000000000E+00
0.000000000000E+00	0.000000000000E+00	0.100000000000E+01
-0.100000000000E+01	0.000000000000E+00	0.000000000000E+00
0.000000000000E+00	0.100000000000E+01	0.000000000000E+00
0.259917552002E+01	0.000000000000E+00	0.259917552002E+01
0.000000000000E+00	-0.100000000000E+01	0.000000000000E+00
0.000000000000E+00	0.000000000000E+00	0.100000000000E+01
0.100000000000E+01	0.000000000000E+00	0.000000000000E+00
0.000000000000E+00	0.259917552002E+01	0.259917552002E+01
0.000000000000E+00	0.100000000000E+01	0.000000000000E+00
0.100000000000E+01	0.000000000000E+00	0.000000000000E+00
0.000000000000E+00	0.000000000000E+00	0.100000000000E+01
0.000000000000E+00	0.000000000000E+00	0.000000000000E+00
0.000000000000E+00	-0.100000000000E+01	0.000000000000E+00
-0.100000000000E+01	0.000000000000E+00	0.000000000000E+00
0.000000000000E+00	0.000000000000E+00	0.100000000000E+01
0.259917552002E+01	0.259917552002E+01	0.000000000000E+00
0.000000000000E+00	-0.100000000000E+01	0.000000000000E+00
0.100000000000E+01	0.000000000000E+00	0.000000000000E+00
0.000000000000E+00	0.000000000000E+00	-0.100000000000E+01
0.259917552002E+01	0.000000000000E+00	0.259917552002E+01
0.000000000000E+00	0.100000000000E+01	0.000000000000E+00
-0.100000000000E+01	0.000000000000E+00	0.000000000000E+00
0.000000000000E+00	0.000000000000E+00	-0.100000000000E+01
0.000000000000E+00	0.259917552002E+01	0.259917552002E+01
0.100000000000E+01	0.000000000000E+00	0.000000000000E+00
0.000000000000E+00	0.000000000000E+00	0.100000000000E+01
0.000000000000E+00	0.100000000000E+01	0.000000000000E+00
0.000000000000E+00	0.000000000000E+00	0.000000000000E+00
0.000000000000E+00	0.000000000000E+00	0.100000000000E+01
0.000000000000E+00	0.100000000000E+01	0.000000000000E+00

```
 0.100000000000E+01  0.000000000000E+00  0.000000000000E+00
 0.000000000000E+00  0.000000000000E+00  0.000000000000E+00
-0.100000000000E+01  0.000000000000E+00  0.000000000000E+00
 0.000000000000E+00  0.000000000000E+00 -0.100000000000E+01
 0.000000000000E+00  0.100000000000E+01  0.000000000000E+00
 0.259917552002E+01  0.259917552002E+01  0.000000000000E+00
-0.100000000000E+01  0.000000000000E+00  0.000000000000E+00
 0.000000000000E+00  0.000000000000E+00  0.100000000000E+01
 0.000000000000E+00 -0.100000000000E+01  0.000000000000E+00
 0.259917552002E+01  0.000000000000E+00  0.259917552002E+01
 0.000000000000E+00  0.000000000000E+00  0.100000000000E+01
 0.000000000000E+00 -0.100000000000E+01  0.000000000000E+00
-0.100000000000E+01  0.000000000000E+00  0.000000000000E+00
 0.000000000000E+00  0.259917552002E+01  0.259917552002E+01
 0.000000000000E+00  0.000000000000E+00 -0.100000000000E+01
 0.000000000000E+00 -0.100000000000E+01  0.000000000000E+00
 0.100000000000E+01  0.000000000000E+00  0.000000000000E+00
 0.259917552002E+01  0.259917552002E+01  0.000000000000E+00
 0.100000000000E+01  0.000000000000E+00  0.000000000000E+00
 0.000000000000E+00  0.000000000000E+00 -0.100000000000E+01
 0.000000000000E+00 -0.100000000000E+01  0.000000000000E+00
 0.000000000000E+00  0.259917552002E+01  0.259917552002E+01
 0.000000000000E+00  0.000000000000E+00 -0.100000000000E+01
 0.000000000000E+00  0.100000000000E+01  0.000000000000E+00
-0.100000000000E+01  0.000000000000E+00  0.000000000000E+00
 0.259917552002E+01  0.000000000000E+00  0.259917552002E+01
  22
 272     0.000000000000     0.000000000000     0.000000000000
 272     2.599175520020     2.599175520020     0.000000000000
 272     0.000000000000     2.599175520020     2.599175520020
 272     2.599175520020     0.000000000000     2.599175520020
 264     5.198351040040     5.198351040040     5.198351040040
 264     2.599175520020     2.599175520020     5.198351040040
 264     5.198351040040     2.599175520020     2.599175520020
 264     2.599175520020     5.198351040040     2.599175520020
   8     3.510724006231     1.299587760010     1.299587760010
   8    -0.911548486211     1.299587760010     1.299587760010
   8     1.299587760010     3.510724006231     1.299587760010
   8     1.299587760010     1.299587760010     3.510724006231
   8     1.299587760010    -0.911548486211     1.299587760010
   8     1.299587760010     1.299587760010    -0.911548486211
   8    -1.299587760010    -3.510724006231    -1.299587760010
   8    -1.299587760010     0.911548486211    -1.299587760010
   8    -3.510724006231    -1.299587760010    -1.299587760010
   8    -1.299587760010    -1.299587760010    -3.510724006231
   8     0.911548486211    -1.299587760010    -1.299587760010
```

8 -1.299587760010 -1.299587760010 0.911548486211
8 3.898763280030 -1.299587760010 -1.299587760010
8 1.299587760010 1.299587760010 -3.898763280030
227 48

Table S2. Mulliken gross atom populations for OP-GHO. Values in parenthesis refer to the cation antisite. The valence populations of the Gd, Hf, and O isolated atoms are 36 *e*, 12 *e*, and 8 e, respectively.

**Atom**	**OP-GHO**	
	**O_{8a} via ghost atoms**	**O_{8a} vacant**
Hf	8.78 (8.86)	8.76 (8.76)
Gd	33.20 (33.34)	33.20 (33.25)
O_{48f}	9.76 (9.63)	9.71 (9.75)
O_{8b}	9.84 (9.54)	9.84 (9.51)
O_{8a}	-0.34 (0.30)	

References

[1] M. Pokhrel, S.K. Gupta, K. Wahid, Y.J.I.c. Mao, Pyrochlore Rare-Earth Hafnate $RE_2Hf_2O_7$ (RE= La and Pr) Nanoparticles Stabilized by Molten-Salt Synthesis at Low Temperature, 58 (2019) 1241-1251.
